# Gene promoter and exon DNA methylation changes in colon cancer development – mRNA expression and tumor mutation alterations

**DOI:** 10.1186/s12885-018-4609-x

**Published:** 2018-06-27

**Authors:** Béla Molnár, Orsolya Galamb, Bálint Péterfia, Barnabás Wichmann, István Csabai, András Bodor, Alexandra Kalmár, Krisztina Andrea Szigeti, Barbara Kinga Barták, Zsófia Brigitta Nagy, Gábor Valcz, Árpád V. Patai, Péter Igaz, Zsolt Tulassay

**Affiliations:** 1 Molecular Medicine Research Group, Hungarian Academy of Sciences, Szentkirályi str 46, Budapest, H-1088 Hungary; 2 2nd Department of Internal Medicine, Semmelweis University, Szentkirályi str 46, Budapest, H-1088 Hungary; 3 Department of Physics of Complex Systems, ELTE Eötvös Loránd University, Pázmány Péter sétány 1/A, Budapest, H-1117 Hungary; 4 Institute of Mathematics and Informatics, Faculty of Sciences, University of Pécs, Ifjúság útja 6, Pécs, H-7624 Hungary

**Keywords:** Colorectal cancer, Adenoma, DNA methylation, Mutation, Methyl capture sequencing, *TP53* signaling pathway

## Abstract

**Background:**

DNA mutations occur randomly and sporadically in growth-related genes, mostly on cytosines. Demethylation of cytosines may lead to genetic instability through spontaneous deamination. Aims were whole genome methylation and targeted mutation analysis of colorectal cancer (CRC)-related genes and mRNA expression analysis of *TP53* pathway genes.

**Methods:**

Long interspersed nuclear element-1 (LINE-1) BS-PCR followed by pyrosequencing was performed for the estimation of global DNA metlyation levels along the colorectal normal-adenoma-carcinoma sequence. Methyl capture sequencing was done on 6 normal adjacent (NAT), 15 adenomatous (AD) and 9 CRC tissues. Overall quantitative methylation analysis, selection of top hyper/hypomethylated genes, methylation analysis on mutation regions and *TP53* pathway gene promoters were performed. Mutations of 12 CRC-related genes (*APC, BRAF, CTNNB1, EGFR, FBXW7, KRAS, NRAS, MSH6, PIK3CA, SMAD2, SMAD4, TP53*) were evaluated. mRNA expression of *TP53* pathway genes was also analyzed.

**Results:**

According to the LINE-1 methylation results, overall hypomethylation was observed along the normal-adenoma-carcinoma sequence. Within top50 differential methylated regions (DMRs), in AD-N comparison *TP73, NGFR, PDGFRA* genes were hypermethylated, *FMN1, SLC16A7* genes were hypomethylated. In CRC-N comparison *DKK2, SDC2, SOX1* genes showed hypermethylation, while *ERBB4, CREB5, CNTN1* genes were hypomethylated. In certain mutation hot spot regions significant DNA methylation alterations were detected. The *TP53* gene body was addressed by hypermethylation in adenomas. *APC*, *TP53* and *KRAS* mutations were found in 30, 15, 21% of adenomas, and in 29, 53, 29% of CRCs, respectively. mRNA expression changes were observed in several *TP53* pathway genes showing promoter methylation alterations.

**Conclusions:**

DNA methylation with consecutive phenotypic effect can be observed in a high number of promoter and gene body regions through CRC development.

**Electronic supplementary material:**

The online version of this article (10.1186/s12885-018-4609-x) contains supplementary material, which is available to authorized users.

## Background

Colorectal cancer (CRC) is a clinically important malignant disease due to its high incidence and mortality. According to the GLOBOCAN estimates with 1.4 million new cases and 694.000 deaths annually, CRC is the third most common cancer in the world, after lung and breast cancers [1].

The majority of sporadic CRCs develop according to the normal-adenoma-dysplasia-carcinoma sequence described by Fearon and Vogelstein [2]. The accumulation of genetic and epigenetic alterations in colonic epithelium leads to CRC through early and late precancerous adenoma stages in which promoter DNA methylation changes of certain tumor suppressor genes with consecutive mRNA expression changes are one of the earliest events, often prior to the appearance of mutations in well-known genes such as the adenomatosis polyposis coli gene (*APC*) [3].

Recently, comprehensive molecular characterization of several human cancers including CRC has been performed and the data integrated into The Cancer Genome Atlas (TCGA) database (https://cancergenome.nih.gov/). Integrative evaluation of genetic, epigenetic and gene expression data of hundreds of CRC and paired normal adjacent tissue (NAT) samples revealed that in addition to the known mutations, epigenetic changes (especially DNA methylation) also play a key role in establishing CRC subtypes with different prognostic and therapeutic phenotypes [4]. The majority (84%) of CRCs were found to be non-hypermutated. Non-hypermutated cancers with distinct colonic or rectal location could be distinguished according to copy-number alteration, DNA methylation or gene expression profiles [4].

DNA methylation changes both in promoter and gene body regions contribute to cancer phenotype as they can affect the gene transcription in several ways [3, 5–7]. In addition to the earlier methods focusing on gene promoter methylation analysis, new technologies, such as BeadChip methylation arrays [4, 8–12], reduced representation bisulfite sequencing (RRBS) [13], whole genomic bisulfite sequencing (WGBS) and methyl capture sequencing (MethylCap-Seq) [7, 14] were applied to study DNA methylation profiles in CRC. While the majority of investigations included CRC and NAT tissues [4, 9, 10, 12, 14], analysis of precancerous adenomas (AD) are represented in a small number of previous studies [8, 11] including a MethylCap-Seq study of WNT pathway genes we undertook [7]. We have also identified hypermethylated markers (mal, T-cell differentiation protein (*MAL*), proline rich membrane anchor 1 (*PRIMA1*), prostaglandin D2 receptor (*PTGDR*) and secreted frizzled related protein 1 (*SFRP1*)) in CRC and adenoma using bisulfite sequencing [15] and determined a common ten-gene methylation signature in colorectal adenomas and CRC based on methylation qPCR arrays [16].

BeadChip 27K and 450K arrays and RRBS offer opportunities for analysis of DNA methylation at single nucleotide resolution mainly within CpG islands, however recently developed Epic BeadChip arrays – besides examination of CpG island methylation - allow more extensive study of CpG sites outside of CpG islands, as well. WGBS provides the most widespread whole methylome analysis at single nucleotide resolution, but it is not commonly used due to its high cost. MethylCap-seq is an alternative genome-wide methylation analysis technique to identify novel differentially methylated regions (DMRs) [17, 18]. It gives extensive information about both promoter and gene body methylation, though at lower resolution [18]. Unlike BeadChip arrays, it is suitable for investigation of mutation hot spot regions within the gene body. It is known that mutations can cause altered DNA methylation and DNA methylation changes also can lead to development of mutations [19, 20]. The mutation rate is higher at methylated CpG sites than non-methylated ones [21, 22]. The change of 5-methylcytosine to thymine via spontaneous deamination [23, 24] ‘which is less effectively repaired by the DNA repair machinery than the cytosine to uracil deamination reaction’ [22, 23] can cause the increased mutability of cytosines within CpG sites.

The aim of this study was to analyze genome-wide tissue DNA methylation differences along the colorectal normal-adenoma-carcinoma sequence progression, including gene body methylation changes using MethylCap-seq. The second aim was to search for a potential relation between DNA methylation and mutation alterations for 12 CRC-associated genes. The possible effects of the genetic and epigenetic changes on neoplastic phenotype at transcriptome level were also examined.

## Methods

### Estimation of global methylation levels using long interspersed nuclear element-1 (LINE-1) bisulfite sequencing

After DNA isolation from 5 colorectal adenoma, 5 CRC and 10 normal (N) colonic biopsy samples, bisulfite conversion of DNA samples was performed using EZ DNA Methylation-Direct Kit (Zymo Research). For quantification of methylation levels of the LINE-1 retrotransposable element, bisulfite-specific PCR (BS-PCR) was done and 146 bp long LINE-1 PCR products were sequenced on Pyromark Q24 system (Qiagen) using the Qiagen Q24 CpG LINE-1 Kit (Qiagen) according to the manufacturers’ instruction.

### MethylCap-seq

Global DNA methylation alterations were determined using MethylCap-seq data of 30 colonic tissue samples (15 AD, 9 CRC, 6 NAT) published previously by our research group [7]. In the previous study [7], only the DNA methylation changes of 160 WNT pathway genes and promoters were evaluated, while in this study whole methylome analysis was performed.

After informed consent of untreated patients, colonic biopsy samples were taken during routine endoscopic intervention. Using parallel formalin-fixed samples from the same site, histological diagnoses were established by experienced pathologists. Tissue samples from untreated CRC patients were also obtained from surgically removed colon or rectal tumors and from NAT that originated from the area farthest available from the tumor. The detailed patient specification has been described earlier [7]. The study was conducted according to the Helsinki declaration and approved by the local ethics committee and government authorities (Regional and Institutional Committee of Science and Research Ethics (TUKEB) Nr.: 69/2008, 202/2009 and 23,970/2011 Semmelweis University, Budapest, Hungary).

Genomic DNA was isolated using High Pure PCR Template Preparation Kit (Roche Applied Science) according to the manufacturer’s instructions [16]. The capture of methylated DNA fragments and next generation sequencing were performed as previously described [7]. Briefly, after fragmentation of 3 μg genomic DNA samples, the DNA fragments with methylated CpGs were selected using the Auto MethylCap kit (Diagenode). Purification of the methylated DNA fraction was carried out on QIAquick PCR purification columns (Qiagen). Library preparation was performed using the TruSeq ChIP Sample Preparation kit (Illumina) and clusters were generated using TruSeq SR Cluster Kit v3-cBot-HS (Illumina). Next generation sequencing of the methylated DNA fragments was performed on the HiScanSQ instrument using TruSeq SBS v3-HS reagents (Illumina,) according to the manufacturer’s instructions. Bowtie2 software with default settings was used to map the 100 bp paired and 50 bp unpaired reads to the hg19 human genome reference assembly [25]. The aligned data were processed using the MEDIPS Bioconductor R package [26]. Methylation probabilities (β-values hereafter) were calculated for 100 bp long analysis windows (differentially methylated regions = DMRs), with respect to genome-wide CpG density dependent Poisson distributions.

### Mutation analysis

Using normal, benign and malignant colorectal tissue samples, mutation hot-spot regions of 12 CRC-associated genes (*APC*, B-Raf proto-oncogene, serine/threonine kinase (*BRAF*), catenin beta 1 (*CTNNB1*), epidermal growth factor receptor (*EGFR*), F-box and WD repeat domain containing 7 (*FBXW7*), KRAS proto-oncogene, GTPase (*KRAS*), NRAS proto-oncogene, GTPase (*NRAS*), mutS homolog 6 (*MSH6*), phosphatidylinositol-4,5-bisphosphate 3-kinase catalytic subunit alpha (*PIK3CA*), SMAD family member 2 and 4 (*SMAD2* and *SMAD4*), tumor protein 53 (*TP53*)) were amplified using a custom-made multiplex PCR panel previously designed by our research group [27]. Amplicon sequencing was carried out on a GS Junior instrument (Roche) using ligated and barcoded adaptors as described earlier [27]. Bead enrichment and sequencing were performed using GS Junior Titanium Sequencing Kit (Roche) according to the Sequencing Method Manual, GS FLX Titanium Series. For variant identification, Amplicon Variant Analyzer software (Roche) was applied.

### Promoter DNA methylation and mRNA expression analysis of *TP53* signaling pathway genes

The list of the *TP53* pathway genes (in total 67 gene symbols) was constructed according to the KEGG pathway database. Promoters were defined as described earlier using Encode ChromHMM results [7]. Promoter DNA methylation was determined using methyl capture results of 30 colonic biopsy samples in a 100 base pair analysis window resolution and DMRs were identified between the diagnostic groups. *In silico* mRNA expression analysis for *TP53* signaling pathway genes was performed using microarray data from colonic tissue samples (Affymetrix HGU133Plus2.0; GEO accession numbers: GSE37364 [28], GSE18105 [29], GSE4107 [30], GSE9348 [31], GSE22242 [32], GSE8671 [33]).

### Statistical analysis

For MethylCap-seq DNA methylation data analysis, differences between diagnostic groups (9 CRC samples versus 6 NAT samples, 15 AD samples versus 6 NAT samples) were characterized by Δβ-values (the differences of the average β-values of each sample group). The top50 candidate DMRs were selected according to the highest absolute values of Δβ-values. For estimation of global methylation levels using LINE-1 bisulfite sequencing, average methylation percentages of 3 analyzed CpG sites were calculated. For gene expression logFC calculations, the differences between the averages of samples groups were compared. During statistical evaluation of DNA methylation and gene expression data, for paired comparisons of diagnostic groups, Student’s t-test and False Discovery Rate (FDR) were applied as the Kolmogorov-Smirnov test resulted in normal distribution and the standard deviation of data were similar. Variance analysis was performed using the non-parametric Kruskal-Wallis test. A *p*-value of < 0.05 was considered as significant.

## Results

### Global DNA methylation alterations of the colorectal normal-adenoma-carcinoma sequence

**Fig. 1:**
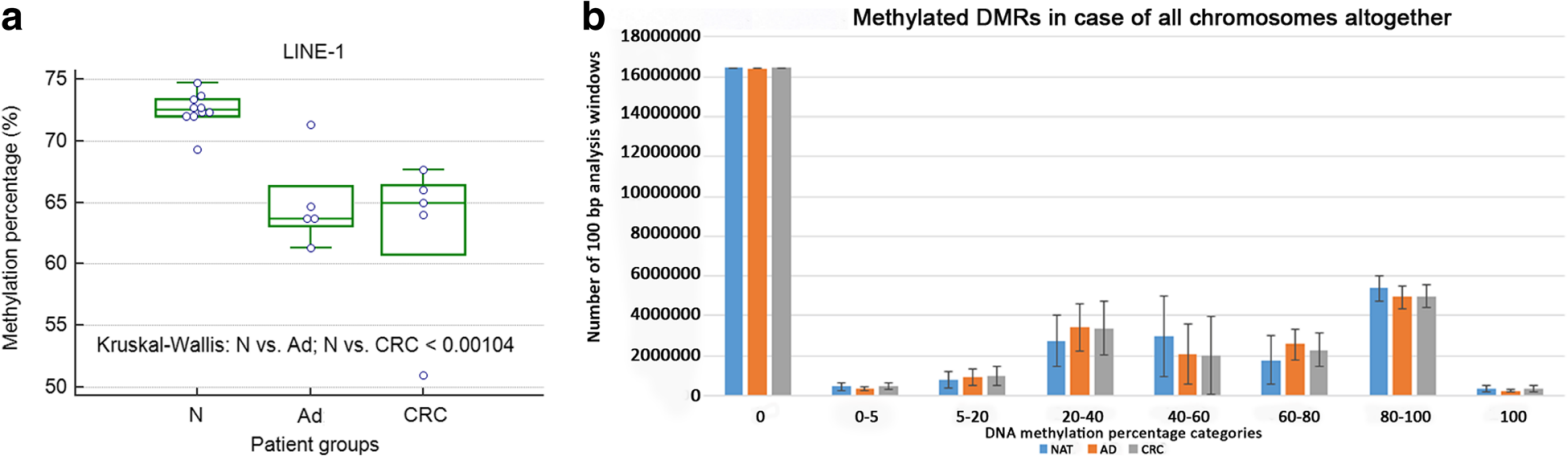
Global DNA methylation alterations of the normal-adenoma-colorectal cancer sequence. **a** DNA methylation of LINE-1 (long interspersed nuclear element-1) in CRC, adenoma and normal tissue samples. N = normal, Ad = adenoma, CRC = colorectal cancer; **b** Category distribution of global DNA methylation in CRC, adenoma and NAT samples analyzed by methyl capture sequencing. DNA methylation percentage categories are shown on the X axis, while the numbers of 100 base pair analysis windows are represented on the Y axis. NAT = normal adjacent tissue, AD = adenoma, CRC = colorectal cancer.

Genome-wide decreases in DNA methylation were observed for samples from the adenoma stage of colorectal carcinogenesis. Based on the LINE-1 bisulfite sequencing results, significant global DNA hypomethylation was detected both in CRC (63 ± 6.7%; *p* = 0.0302) and adenoma samples (65 ± 3.8%; *p* = 0.0093) compared to normal tissue (73 ± 1.4%). Variance analysis also revealed significantly lower DNA methylation level both in CRC and adenoma than in normal samples (Kruskal-Wallis test: *p* < 0.00104) (Fig. 1a). MethylCap-seq results showed that decreased DNA methylation appeared principally in 40–60% and 80–100% methylation percentage categories in adenoma and CRC samples compared to NAT controls (Fig. 1b).

### Top DMRs in CRC and adenoma samples identified by MethylCap-seq

In CRC samples known CRC-associated genes including heparan sulfate-glucosamine 3-sulfotransferase 2 (*HS3ST2*), dickkopf WNT signaling pathway inhibitor 2 (*DKK2*), tissue factor pathway inhibitor 2 (*TFPI2*) and syndecan 2 (*SDC2*) occurred in the top50 significantly hypermethylated 100 base paired regions (*p* < 0.001), showing elevated promoter DNA methylation levels located within CpG islands. Δβ-values representing methylation differences between CRC and NAT samples were in a range from 0.68 to 0.81. More than one third of the top50 hypermethylated DMRs align with weak (9%) or active (9%) promoters according to the Encode ChromHMM data. The majority of the top50 DMRs that were significantly hypomethylated in CRC compared to NAT samples (*p* < 0.001) could not be assigned to genes, gene promoters, and were located in intergenic regions. Similar to the hypermethylated DMRs, large differences were found for hypomethylated DMRs with Δβ-values between − 0.74 and − 0.65 (Additional file 1: Table S1A, B).

In the AD versus NAT comparison, 94% of the top50 highly methylated DMRs were found in CpG islands including generally known CRC-associated DNA methylation markers like Fli-1 proto-oncogene, ETS transcription factor (*FLI1*), GATA binding protein 4 (*GATA4*) and nerve growth factor receptor (*NGFR*). The top50 significant (*p* < 0.0001) methylation alterations appeared to be more intensive in adenomas compared to NAT samples (Δβ-values were between 0.86 and 0.79). Considering Encode ChromHMM data, 38% of top50 hypermethylated DMRs were found to be located in promoter regions, and 26% can function as active promoters. Similar to the results in CRC versus NAT comparison, almost all of the top50 DMRs showing significantly decreased DNA methylation in AD could not be annotated (*p* < 0.0001) with stronger methylation differences than found in CRC versus NAT (Δβ-values between − 0.90 and − 0.74) (Additional file 1: Table S1C, D).

### DNA methylation alterations and expression of CRC-associated, frequently mutated genes

**Fig. 2:**
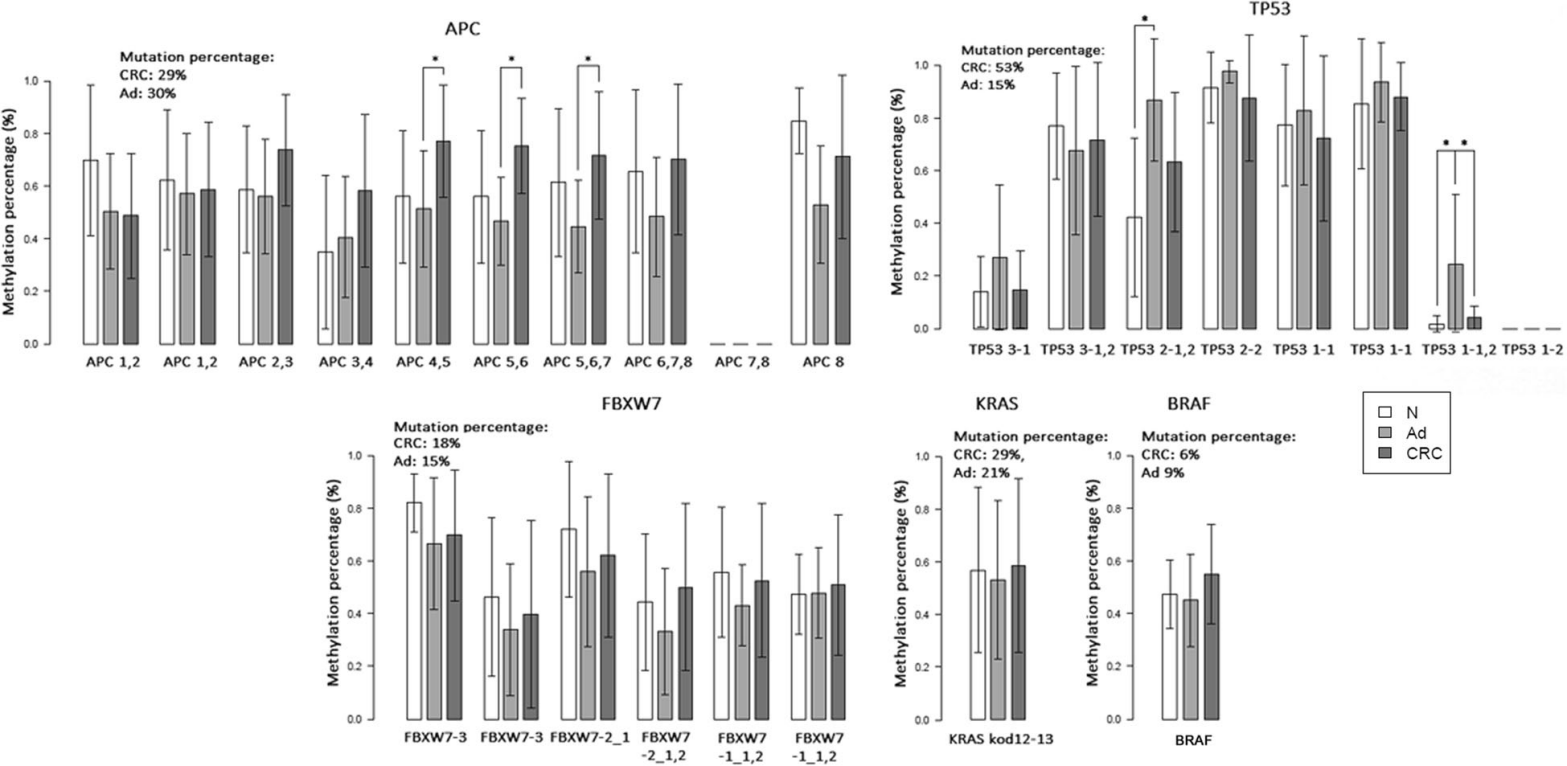
DNA methylation alterations in mutation hot-spot regions of genes frequently mutated in CRC and adenoma. Methylation percentage values are shown in 100 base pair analysis regions located in mutation hot-spot areas of genes (*TP53, APC, KRAS, BRAF* and *FBXW7*) frequently mutated in CRC and adenoma tissue. The frequencies of mutations in CRC and adenoma samples detected in our previous multiplex PCR-based CRC mutation hot-spot sequencing study [27] are also represented. *p < 0.05, CRC = colorectal cancer, Ad = adenoma, N = normal adjacent tissue

**Fig. 3:**
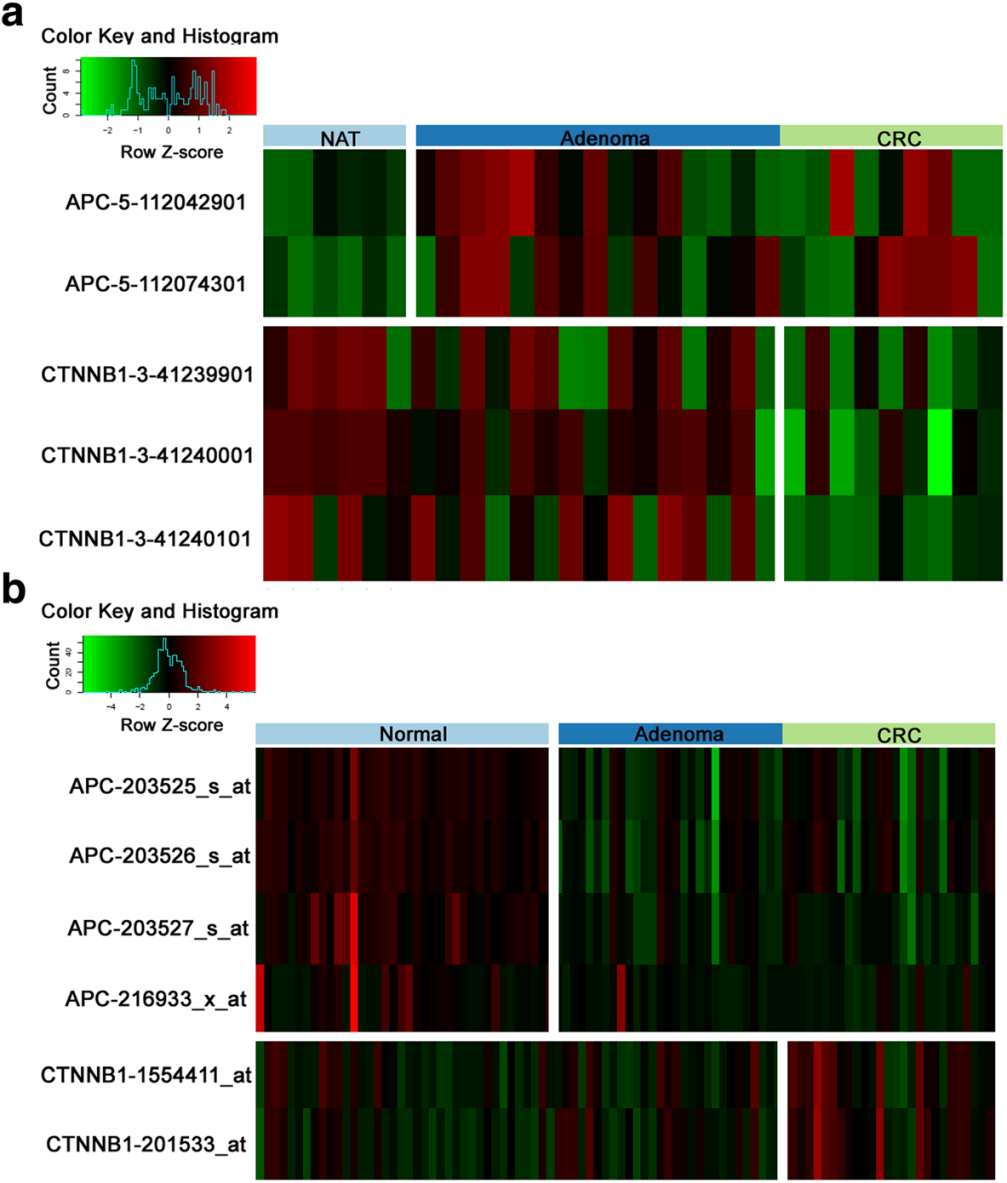
Inverse promoter DNA methylation and mRNA expression alterations of *APC* and *CTNNB1* genes in CRC and adenoma samples. **a** Significant DMRs in promoter regions of *APC* and *CTNNB1* genes in CRC and adenoma samples (p < 0.05). Hypermethylation is marked with red, while hypomethylated DMRs are green. The names of the DMRs indicate the official gene symbol_number of the chromosome_start position of the DMR. CRC = colorectal cancer, NAT = normal adjacent tissue. **b** mRNA expression pattern of *APC* and *CTNNB1* genes in CRC and adenoma (GSE37364 [28]). Overexpression is marked with red, while downregulated genes are green. CRC = colorectal cancer

The mutation frequencies of a panel consisting 12 CRC-associated genes in CRC and AD samples were measured in our previous multiplex PCR-based CRC mutation hot-spot sequencing study [27]. DNA methylation alterations were also detected in the mutation hot-spot regions of 12 analyzed CRC-associated genes that are frequently mutated, including *TP53*, *APC*, *KRAS*, *BRAF* and *FBXW7*. DNA methylation changes on 100 base pair long analysis windows located on mutation hot-spot regions of *TP53*, *APC*, *KRAS*, *BRAF* and *FBXW7* can be seen in Fig. 2. Evaluation of promoter methylation patterns of the 12 frequently mutated genes revealed several significant alterations including hypermethylation of the *APC* promoter in CRC and AD tissue specimens (*p* < 0.05; Δβ = 0.27–0.39) (Fig. 3a), hypermethylation of the *TP53* promoter in AD (*p* < 0.001; Δβ = 0.40) and hypomethylation of *CTNNB1* (*p* < 0.05; Δβ between − 0.30 and − 0.45) (Fig. 3a) and *SMAD2* (*p* = 0.024; Δβ = − 0.28) in CRC compared to NAT samples. *SMAD4* promoter region was found to be hypomethylated both in AD and CRC biopsy samples (p < 0.05; Δβ between − 0.25 and − 0.32). mRNA expression profiles of the 12 analyzed CRC-associated genes revealed that *APC* and *CTNNB1* could be regulated by DNA methylation during the colorectal carcinogenesis as showing inverse relation between promoter DNA methylation and mRNA expression (Fig. 3b) .

### DNA methylation on *TP53* signaling pathway gene promoters – Relation with gene expression results

**Fig. 4:**
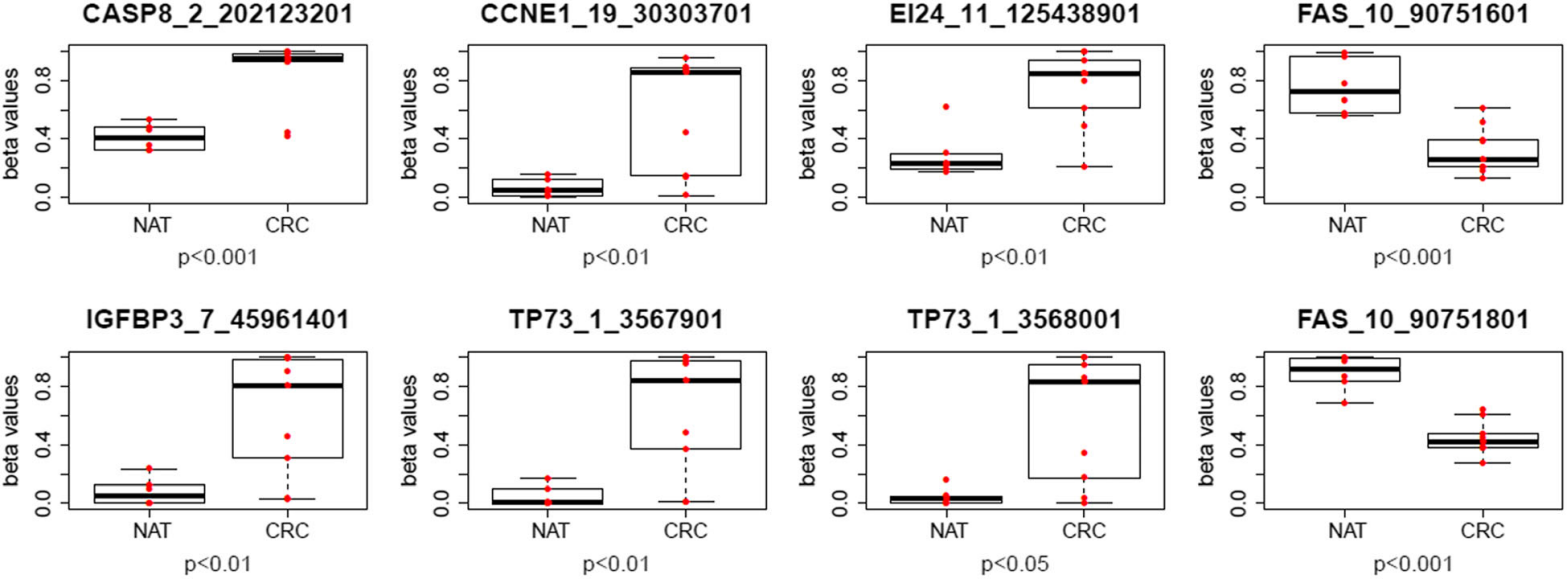
Box plots of DMRs in *TP53* pathway gene promoters with the highest DNA methylation differences between CRC and NAT samples. Box plots represent the DNA methylation levels (β-values) of differentially methylated regions (DMRs) in *TP53* pathway gene promoters showing the highest DNA methylation differences between CRC and NAT tissue samples. Individual DNA methylation level values are shown by red dots, and the median and standard deviation of the β-values are also demonstrated. The names of the DMRs indicate the official gene symbol_number of the chromosome_start position of the DMR. NAT = normal adjacent tissue, CRC = colorectal cancer, CASP8 = caspase 8, CCNE1 = cyclin E1, EI24 = EI24 autophagy associated transmembrane protein, FAS = Fas cell surface death receptor, IGFBP3 = insulin-like growth factor binding protein 3, TP73 = tumor protein p73

The *TP53* pathway genes selected according to the KEGG pathway database were represented with 67 gene symbols. Promoters were defined as described earlier using Encode ChromHMM results [7]. In the CRC versus NAT comparison, 26.9% of *TP53* pathway genes (18 from 67 genes) showed significant DNA methylation alterations in their promoter regions with at least a 10% methylation difference (*p* < 0.05, Δβ ≥ 0.1) (Table 1). In CRC samples hypermethylated DMRs were found in the promoter regions of 11 genes such as caspase 8 (*CASP8*), cyclin dependent kinase inhibitor 1A and 2A (*CDKN1A* and *CDKN2A*), insulin-like growth factor binding protein 3 (*IGFBP3*), sestrin 2 (*SESN2*) and tumor protein p73 (*TP73*), while seven *TP53* pathway genes including G2 and S-phase expressed 1 (*GTSE1*) showed hypomethylation in their promoters. The box plots of the significant hyper-, and hypomethylated DMRs in *TP53* pathway gene promoters showing the highest DNA methylation differences between CRC and NAT samples are represented on Fig. 4 and the box plots of all DMRs fulfilling the criteria can be seen in Additional file 2: Figure S1.

**Table 1:** DNA methylation alterations in promoter regions of *TP53* signaling pathway genes in CRC and AD tissues compared to NAT samples

Gene symbol	Gene name	chr	start	stop	Δβ (CRC-NAT)	Δβ (AD-NAT)
*ATM*	ATM serine/threonine kinase	11	108092901	108093000		0.22*
*ATR*	ATR serine/threonine kinase	3	142298701	142298800	0.26*	
*BAX*	BCL2 associated X, apoptosis regulator	19	49457601	49457700		−0.20*
*BBC3*	BCL2 binding component 3	19	47734901	47735000		0.29*
			47735001	47735100		0.24*
			47735601	47735700		−0.17*
*CASP8*	caspase 8	2	202098501	202098600		0.31**
			202098601	202098700		0.26*
			202122001	202122100		0.21*
			202122101	202122200		0.32*
			202123101	202123200	0.27*	
			202123201	202123300	0.43***	
*CCND3*	cyclin D3	6	41908401	41908500		−0.20*
			42016801	42016900		0.21*
			42016901	42017000		0.27*
*CCNE1*	cyclin E1	19	30303701	30303800	0.51**	
*CDK1*	cyclin dependent kinase 1	10	62539101	62539200		−0.25*
			62539201	62539300		−0.33**
*CDK6*	cyclin dependent kinase 6	7	92466601	92466700		0.33**
			92466701	92466800		0.29*
			92466801	92466900		0.26*
*CDKN1A*	cyclin dependent kinase inhibitor 1A	6	36644601	36644700	0.23*	
*CDKN2A*	cyclin dependent kinase inhibitor 2A	9	21975301	21975400	0.29*	
			21993901	21994000		0.25*
			21994001	21994100		0.37*
*CHEK1*	checkpoint kinase 1	11	125496401	125496500		0.23*
			125496501	125496600		0.42**
			125496601	125496700		0.32*
*CYCS*	cytochrome c, somatic	7	25164001	25164100		0.32*
			25164201	25164300		0.33*
			25165801	25165900	0.17*	
*DDB2*	damage specific DNA binding protein 2	11	47237401	47237500		−0.28*
			47237501	47237600		−0.29*
*EI24*	EI24, autophagy associated transmembrane protein	11	125438801	125438900	0.29*	
			125438901	125439000	0.46**	
			125439001	125439100	0.36*	
*FAS*	Fas cell surface death receptor	10	90751601	90751700	−0.43***	
			90751701	90751800	−0.22*	
			90751801	90751900	−0.45***	−0.27*
			90751901	90752000	−0.26*	
*GADD45A*	growth arrest and DNA damage inducible alpha	1	68150801	68150900		0.30*
*GTSE1*	G2 and S-phase expressed 1	22	46693501	46693600	−0.26*	−0.31**
			46693601	46693700		−0.33**
*IGF1*	insulin like growth factor 1	12	102871901	102872000	−0.26*	−0.23*
*IGFBP3*	insulin like growth factor binding protein 3	7	45961101	45961200		0.46*
			45961401	45961500	0.54**	0.73***
			45961501	45961600	0.48*	0.60***
			45961701	45961800		0.42**
*MDM4*	MDM4, p53 regulator	1	204486501	204486600		0.14*
*PTEN*	phosphatase and tensin homolog	10	89621101	896212200	−0.34*	
*RCHY1*	ring finger and CHY zinc finger domain containing 1	4	76439201	76439300		−0.23*
			76440301	76440400		−0.29*
*RFWD2*	ring finger and WD repeat domain 2	1	176177801	176177900	−0.33*	
*RRM2*	ribonucleotide reductase regulatory subunit M2	2	10261301	10261400	0.32*	
*SERPINE1*	serpin family E member 1	7	100773001	100773100		0.24*
*SESN1*	sestrin 1	6	109331401	109331500		0.27*
			109416501	109416600		0.11*
*SESN2*	sestrin 2	1	28585601	28585700	0.24*	
*SESN3*	sestrin 3	11	94965001	94965100	−0.24*	
*SFN*	stratifin	1	27189901	27190000		−0.35*
			27190301	27190400		−0.34*
*SHISA5*	shisa family member 5	3	48513701	48513701		0.24*
*THBS1*	thrombospondin 1	15	39871901	39872000	−0.27*	
			39872801	39872900		−0.22*
*TP73*	tumor protein p73	1	3567901	3567901	0.58**	0.82***
			3568001	3568001	0.53*	0.81***
			3568101	3568101		0.26*
*TSC2*	TSC complex subunit 2	16	2097201	2097300		−0.26*

*p* < 0.05 *; *p* < 0.01 **; *p* < 0.001 ***

By applying the same criteria, significant promoter DNA methylation changes were observed in 37.3% of *TP53* pathway genes (25/67) in AD compared to NAT samples (p < 0.05, Δβ ≥ 0.1) (Table 1). Fifteen *TP53* pathway genes showed elevated promoter methylation in AD samples including *CDKN2A*, *IGFBP3* and *TP73*, while hypomethylation was detected in the promoter regions of 10 genes such as *GTSE1*, damage specific DNA binding protein 2 (*DDB2*) and cyclin dependent kinase 1 (*CDK1*).

Using *in silico* expression analysis of microarray data from colonic biopsy samples (Affymetrix HGU133Plus2.0 GEO accession numbers: GSE37364 [28], GSE18105 [29], GSE4107 [30], GSE9348 [31], GSE22242 [32], GSE8671 [33]), an inverse relation between promoter DNA methylation alteration and mRNA expression (Table 2) was shown for a number of differentially methylated TP53 pathway genes including *CDKN1A*, *CDKN2A*, *GTSE1*, *IGFBP3*, *SESN2* and *SESN3* (Table 2).

**Table 2:** *TP53* signaling pathway genes showing inverse relation between promoter DNA methylation and mRNA expression

Gene symbol	Gene name	promoter DNA methylation in our Metcap-Seq study*	mRNA expression in tumor/adenoma versus normal (logFC_min_ and logFC_max_)**	Alterations in cancer/CRC/adenoma***
*BAX*	BCL2 associated X, apoptosis regulator	hypomethylated in adenoma	0.36 and 1.09	- downregulation in CRC [72]
*CDK1*	cyclin dependent kinase 1	hypomethylated in adenoma	0.42 and 2.12	- overexpression in cancer [73]
*CDKN1A* (p21)	cyclin dependent kinase inhibitor 1A	hypermethylated in CRC	−2.16 and − 0.67	- loss of expression in CRC [74]
*CDKN2A* (p16)	cyclin dependent kinase inhibitor 2A	hypermethylated in CRC hypermethylated in adenoma	−0.81	- hypermethylated in CRC [75, 76] and in adenoma [76]
*CYCS*	cytochrome c, somatic	hypermethylated in CRC hypermethylated in adenoma	−0.43 and − 1.83; − 0.33 and − 1.27	- loss of protein expression in CRC and in adenoma [77] - its downregulation correlates with apoptosis resistance [77]
*DDB2*	damage specific DNA binding protein 2	hypomethylated in adenoma	1.31 and 1.54	- suppresses tumorigenicity [78] - reduces invasiveness of CRC [79] - downregulated in high-grade CRC [79]
*GTSE1*	G2 and S phase expressed	hypomethylated in CRC hypomethylated in adenoma	0.27 and 2.61	- upregulation in several tumor types [80, 81]
*IGFBP3*	insulin like growth factor binding protein 3	hypermethylated in CRC hypermethylated in adenoma	−1.11 and − 2.27	- hypermethylated in lung cancer [82] - downregulation in esophageal carcinoma [83] - lower level is associated with increased colon adenoma risk [84]
*SESN2*	sestrin 2	hypermethylated in CRC	−1.86 and − 0.72	- downregulation in tumors [85–87] - predicts unfavorable CRC outcome [85]
*SESN3*	sestrin 3	hypomethylated in CRC	0.46 and 1.09	- involved in in vitro resistance to Irinotecan [88]

*Δβ-values see in Table 1

**according to Affymetrix HGU133Plus2.0 microarray data (GEO accession numbers: GSE37364 [28], GSE18105 [29], GSE4107 [30], GSE9348 [31], GSE22242 [32], GSE8671 [33])

***according to previous literature data

## Discussion

The accumulation of DNA methylation alterations accompanied by genetic changes such as mutations and deletions is known to contribute to the pathogenesis of various cancer types including CRC [3, 4]. Comprehensive DNA methylation changes found in precancerous adenoma stages can serve as early detection markers [7, 8, 11, 34]. In this study, global DNA methylation alterations were analyzed along the colorectal normal-adenoma-carcinoma sequence, and top differentially methylated genes/regions were identified using genome-wide MethylCap-seq analysis. The second aim of the study was to find out if there is a potential correlation between DNA methylational and mutational alterations for 12 CRC-associated genes. Furthermore, the possible effects of the genetic and epigenetic changes on *TP53* signaling pathway genes at the transcriptome level were also examined.

Global hypomethylation was detected by LINE-1 bisulfite sequencing in CRC samples compared to normal tissue in line with previous data [35–37]. Although to a lower extent, global DNA hypomethylation could be detected as early as the AD stage. LINE-1 bisulfite sequencing was used for overall hypomethylation analysis due to its superior advance over MethylCap-seq, which predominantly targets genomic regions with high methylated CpGs density [14].

In this study, we identified 22 novel AD- and/or CRC-associated hypermethylated DMRs (approximately one fourth of top50 hypermethylated DMRs) which could be assigned to genes with previously undescribed methylation changes in cancers including CRC. These markers are principally involved in transcription regulation (e.g. *BHLHE23*, *CUX2*, *HLX*, *MAFB*, *MKX*, *NKX1–1,* and *GSC2*), transport processes (e.g. *SLC24A2*, *GLRA3*, *LRRC38*, *SNAP91*), and intracellular signaling (e.g. *RGS20*, *GNAL*, *NRG3*). Among the hypermethylated transcription factors, the expression of H2.0 like homeobox (*HLX*) was found to be reduced in moderately differentiated CRCs [38]. Moreover, *HLX* is also considered as a tumor suppressor in hepatocellular carcinoma [39]. The platelet derived growth factor receptor alpha (*PDGFRA*) was observed to be hypermethylated in AD compared to normal controls in our study. It was found to be overexpressed in CRC, but - in accordance with promoter hypermethylation detected in our MethylCap-seq study – it was down-regulated in adenomatous polyps [40]. Nevertheless, one fifth of hypermethylated and the majority of hypomethylated DMRs could not be associated with known genes, both in CRC versus NAT and AD versus NAT comparisons. The identified significant top50 methylation changes could be observed in a high proportion (> 80%) of the specimens within a sample group compared to the mutational alterations analyzed in this study.

On the basis of the methylation levels of the top50 hypomethylated and hypermethylated markers determined in this study, including the newly identified DMRs, the clear separation of CRC and NAT samples was also apparent for an independent sample set (Additional file 2: Figure S2). Furthermore, a partially overlapping set of samples also showed consistent DNA methylation profiles analyzed by MethylCap-seq and EpiTect Methyl qPCR methods (Additional file 2: Figure S3).

Approximately half of the top50 identified hypermethylated DMRs in CRC represent genes found to demonstrate elevated DNA methylation levels in different types of cancers [14, 16, 34, 41–60]. Seven of the top50 markers (*BNC1* [16], *DKK2* [48–50], *HS3ST2* [51], *MIR124–3* [52], *SDC2* [14, 34, 53, 54], *TFPI2* [55, 56] and *ZIC1* [57]) were previously described as methylated genes in CRC. Hypermethylation of basonuclin (*BNC1*) zinc finger protein, *SDC2* transmembrane heparin sulfate proteoglycan, and *DKK2* dickkopf WNT signaling pathway inhibitor 2 genes were also reported in previous studies by our research group [16, 34]. Among the annotated AD versus NAT top50 hypermethylated DMRs, several markers were found to be hypermethylated in various cancers including *FLI1* [58, 59], *GATA4* [51] and *NGFR* [60]. These showed elevated methylation levels in CRC samples in other studies.

In this study, we present a comparative analysis between the promoter methylation and mRNA expression data of 12 genes frequently mutated during colorectal carcinogenesis and progression. The results revealed that DNA methylation can play a role in the regulation of *APC* and *CTNNB1* expression in addition to and in parallel with the mutational changes. The above genes are members of the WNT signaling pathway investigated in details in our previous analysis [7]. Hypermethylation of the *APC* promoter [7, 50, 61, 62] and hypomethylation of the *CTNNB1* promoter [7, 49] in AD and CRC samples have also been detected in other studies indicating that the DNA methylation alterations of frequently mutated canonical WNT pathway key genes can contribute to its constitutive activation in colorectal carcinogenesis from the premalignant adenoma stage.

Farkas et al. evaluated DNA methylation changes of genes frequently mutated in CRC using BeadChip450K technology, including 11 of the 12 genes analyzed in our study [49] and reported hypomethylation in *CTNNB1* and *SMAD2* promoters in CRC compared to NAT samples. Decreased promoter DNA methylation levels of these genes were also observed in our MethylCap-seq analysis together with methylation alterations of other genes such as *SMAD4* and *TP53* promoters during colorectal carcinogenesis.

In the current project, DNA methylation alterations were also detected in the mutation hot-spot regions of 12 analyzed CRC-associated frequently mutated genes including *TP53, APC, KRAS, BRAF,* and *FBXW7*. In accordance with the observation that C - T transitions at CpG sites are the most prevalent mutations in *TP53* gene in colon tumors [63], the high mutation rate and methylation changes at mutation hot spot regions of this gene could be detected in our study. DNA methylation can cause mutations in tumor suppressor genes such as *TP53*, as mutations occur 10–40 times more frequently on the basis of methylated cytosine than of unmethylated cytosine [19, 20]. The conversion of 5-methylcytosine to thymine via spontaneous deamination [23, 24] or by the APOBEC/AID system [64] can lead to a high mutational burden of 5-methylcytosine. The 5-methylcytosine can be involved in increased mutability through other mechanisms. According to a recent report, elevated C to G transversion rate in cancer genomes can be associated with 5-hydroxymethylcytosines derived by the oxidation of 5-methylcytosine catalyzed by TET proteins [65]**.**

Hypomethylation was also detected in addition to the elevated methylation levels on certain mutation hot-spots. This is only seemingly contradictory to previous data indicating that the mutation rate is higher on methylated CpG sites than on unmethylated ones [21], as the relative hypomethylation (from high level to intermediate level) and not the absolute loss of DNA methylation was observed on certain mutation hot-spots in our study. It is in conjunction with the results of a recent work describing that among the methylated CpG sites, the rate of mutations (or SNP density) was found to be increased on less methylated CpG sites (20–60%) as compared to high-intermediately and highly methylated CpGs (60–80%; > 80%) [21, 66]. Cancer-associated overall hypomethylation of the genome including heterochromatic DNA repeats, retrotransposons, and endogenous retroviral elements also contribute to genome instability [20].

In our analysis, DMRs could be identified on all chromosomes with the relatively largest number of aligned sequence reads on chromosome 17, similar to the MethylCap-seq study performed by Simmer et al. [14]. Next, DNA methylation alterations of *TP53* (encoded on chr 17) signaling pathway genes were also investigated. *TP53* pathway deregulation frequently occurs through the mutations or deletion of *TP53* itself [67]. Outside the mutations of the *TP53* gene, this pathway is rarely hit by any other mutations/polymorphisms [68–70]. Other mechanisms, such as epigenetic regulation including DNA methylation changes of *TP53* pathway genes, also contribute to attenuating the pathway and participate in cancer development [67], and *TP53* itself is also thought to regulate cancer-associated genes showing altered methylation patterns [71]. Accordingly, our MethylCap-Seq analysis revealed significant promoter DNA methylation changes in approximately one third of *TP53* signaling pathway genes in CRC. Moreover, an even greater proportion of *TP53* pathway gene promoters (around 40%) showed altered DNA methylation in AD samples compared to NAT controls. The alterations of the identified *TP53* pathway genes with inverse promoter DNA methylation and mRNA expression differences (Table 2) were found to be associated with tumorigenesis in different cancer types including CRC [72–88]. Among these markers, in addition to the down-regulation of well known p16 (*CDKN2A*) [75, 76] and p21 (*CDKN1A*) [74] cyclin dependent kinase inhibitors, BCL2 associated X, apoptosis regulator (*BAX*) [72], *SESN2* [85–87], *IGFBP3* [84] and cytochrome c, somatic (*CYCS*) [77] are also thought to exert tumor suppressor functions. Diminished or loss of CYCS protein expression in AD and CRC tissue was found to be correlated with apoptosis resistance [77]. *DDB2* damage specific DNA binding protein, which was described to suppress the tumorigenicity in case of ovarian cancer [78] and reduces CRC invasiveness [79], showed promoter hypomethylation and overexpression in AD samples in our study, suggesting its contribution to the inhibition of uncontrolled expansion in the adenoma stage.

## Conclusions

Using genome-wide DNA methylation analysis, we identified novel aberrant methylation profiles of genes including *HLX*, *CUX2*, *MKX*, *NRG3* and *PDGFRA* associated with the colorectal adenoma-carcinoma sequence progression. In addition to the genetic changes, DNA methylation alterations were also shown in the mutation hot-spot regions of 12 analyzed, CRC-associated, frequently mutated genes including, *TP53, APC, KRAS, BRAF,* and *FBXW7*. Global hypomethylation – which might be linked to genetic instability - could be detected as early as the adenoma stage.

Our study also revealed that promoter DNA methylation changes influence the mRNA expression level in the case of a significant part of the *TP53* pathway genes. Thus epigenetic alterations can also contribute to a whole pathway-related effect on DNA repair and apoptosis in addition to single gene (e.g. *TP53*) mutations.

In summary, the methyl-capture sequencing technique yielded reproducible, clinically relevant results on the whole genome level which are related to cancer phenotype development through mRNA expression changes and to the cancer genotype through the link of mutation formation.

## Additional files

Additional file 1: **Table S1A.** Top50 hypermethylated DMRs in CRC versus NAT comparison. **Table S1B.** Top50 hypomethylated DMRs in CRC versus NAT comparison. **Table S1C.** Top50 hypermethylated DMRs in adenoma tissue compared to NAT samples. **Table S1D.** Top50 hypomethylated DMRs in adenoma tissue compared to NAT samples. (DOC 253 kb)

Additional file 2: **Figure S1.** Box plots of significant DMRs in *TP53* pathway gene promoters between CRC and NAT samples. **Figure S2.** DNA methylation pattern of top50 hypermethylated DMRs on an independent set of samples. **Figure S3.** DNA methylation profiles of the overlapping regions on methyl capture sequencing and EpiTect array results. (DOC 902 kb)

## Abbreviations

AD: Adenoma
AID: Activation-induced cytidine deaminase
APC: Adenomatosis polyposis coli
APOBEC: Apolipoprotein B mRNA editing enzyme catalytic subunit
BAX: BCL2 associated X, apoptosis regulator
BHLHE23: Basic helix-loop-helix family member e23
BNC1: Basonuclin
BRAF: B-Raf proto-oncogene, serine/threonine kinase
BS-PCR: Bisulfite-specific polymerase chain reaction
CASP8: Caspase 8
CDK1: Cyclin dependent kinase 1
CDKN1A and CDKN2A: Cyclin dependent kinase inhibitor 1A and 2A
CNTN1: Contactin 1
CRC: Colorectal cancer
CREB5: CAMP responsive element binding protein 5
CTNNB1: Catenin beta 1
CUX2: Cut like homeobox 2
CYCS: Cytochrome c, somatic
DDB2: Damage specific DNA binding protein 2
DKK2: Dickkopf WNT signaling pathway inhibitor 2
DMR: Differentially methylated regions
EGFR: Epidermal growth factor receptor
ERBB4: Erb-b2 receptor tyrosine kinase 4
FBXW7: F-box and WD repeat domain containing 7
FDR: False Discovery Rate
FLI1: Fli-1 proto-oncogene, ETS transcription factor
FMN1: Formin 1
GATA4: GATA binding protein 4
GEO: Gene Expression Omnibus database
GLRA3: Glycine receptor alpha 3
GNAL: G protein subunit alpha L
GSC2: Goosecoid homeobox 2
GTSE1: G2 and S-phase expressed 1
HLX: H2.0 like homeobox
HS3ST2: Heparan sulfate-glucosamine 3-sulfotransferase 2
IGFBP3: Insulin-like growth factor binding protein 3
KRAS: KRAS proto-oncogene, GTPase
LINE-1: Long interspersed nuclear element-1
LRRC38: Leucine rich repeat containing 38
MAFB: MAF bZIP transcription factor B
MAL: Mal, T-cell differentiation protein
MethylCap-Seq: Methyl capture sequencing
MIR124–3: MicroRNA 124–3
MKX: Mohawk homeobox
MSH6: MutS homolog 6
N: Normal tissue
NAT: Normal adjacent tissue
NGFR: Nerve growth factor receptor
NKX1–1: NK1 homeobox 1
NRAS: NRAS proto-oncogene, GTPase
NRG3: Neuregulin 3
PDGFRA: Platelet derived growth factor receptor alpha
PIK3CA: Phosphatidylinositol-4,5-bisphosphate 3-kinase catalytic subunit alpha
PRIMA1: Proline rich membrane anchor 1
PTGDR: Prostaglandin D2 receptor
RGS20: Regulator of G protein signaling 2
RRBS: Reduced representation bisulfite sequencing
SDC2: Syndecan 2
SESN2: Sestrin 2
SFRP1: Secreted frizzled related protein 1
SLC16A7: Solute carrier family 16 member 7
SLC24A2: Solute carrier family 24 member 2
SMAD2 and SMAD4: SMAD family member 2 and 4
SNAP91: Synaptosome associated protein 91
SOX1: SRY-box 1
TCGA: The Cancer Genome Atlas
TET: Tet methylcytosine dioxygenase
TFPI2: Tissue factor pathway inhibitor 2
TP53: Tumor protein p53
TP73: Tumor protein p73
WGBS: Whole genomic bisulfite sequencing
ZIC1: Zic family member 1
