# Supplementary material for: Gene promoter and exon DNA methylation changes in colon cancer development – mRNA expression and tumor mutation alterations
Source: BMC Cancer. 2018 Jun 27;18:695. doi: 10.1186/s12885-018-4609-x (PMC6020382; doi:10.1186/s12885-018-4609-x)
Supplement: Supplementary file 1 — Table S1A. Top50 hypermethylated DMRs in CRC versus NAT comparison. Table S1B. Top50 hypomethylated DMRs in CRC versus NAT comparison. Table S1C. Top50 hypermethylated DMRs in adenoma tissue compared to NAT samples. Table S1D. Top50 hypomethylated DMRs in adenoma tissue compared to NAT samples. (DOC 253 kb) [file 12885_2018_4609_MOESM1_ESM.doc]

**Table S1A. Top50 hypermethylated DMRs in CRC versus NAT comparison**

| **chr** | **start** | **stop** | **gene symbol** | **p value** | **Δβ**  **(CRC-NAT)** |
| --- | --- | --- | --- | --- | --- |
| chr3 | 58572601 | 58572700 | FAM107A | 6.21 x 10^-6^ | 0.81 |
| chr7 | 157486001 | 157486100 | PTPRN2 | 7.38 x 10^-7^ | 0.78 |
| chr10 | 106400501 | 106400600 | SORCS3 | 1.21 x 10^-5^ | 0.77 |
| chr1 | 17019401 | 17019500 | ESPNP | 6.41 x 10^-5^ | 0.76 |
| chr1 | 229567501 | 229567600 | ACTA1 | 1.25 x 10^-8^ | 0.76 |
| chr14 | 48143601 | 48143700 | MDGA2 | 3.71 x 10^-7^ | 0.76 |
| chr6 | 6002501 | 6002600 | NRN1 | 1.83 x 10^-7^ | 0.75 |
| chr4 | 1398101 | 1398200 | NA | 2.58 x 10^-5^ | 0.75 |
| chr13 | 112722301 | 112722400 | SOX1 | 1.43 x 10^-4^ | 0.74 |
| chr2 | 242481401 | 242481500 | NA | 1.83 x 10^-4^ | 0.74 |
| chr3 | 58572501 | 58572600 | FAM107A | 2.44 x 10^-5^ | 0.74 |
| chr20 | 39317001 | 39317100 | MAFB | 2.61 x 10^-4^ | 0.73 |
| chr20 | 39317101 | 39317200 | MAFB | 3.61 x 10^-4^ | 0.72 |
| chr16 | 22825001 | 22825100 | HS3ST2 | 2.65 x 10^-4^ | 0.72 |
| chr4 | 107956901 | 107957000 | DKK2 | 1.44 x 10^-5^ | 0.72 |
| chr3 | 36805401 | 36805500 | NA | 3.16 x 10^-7^ | 0.72 |
| chr2 | 29337901 | 29338000 | CLIP4 | 1.15 x 10^-5^ | 0.72 |
| chr3 | 147127601 | 147127700 | ZIC1 | 4.17 x 10^-5^ | 0.71 |
| chr15 | 83952401 | 83952500 | BNC1 | 1.44 x 10^-4^ | 0.71 |
| chr16 | 86612401 | 86612500 | FOXL1 | 1.12 x 10^-5^ | 0.71 |
| chr13 | 28498501 | 28498600 | PDX1 | 1.82 x 10^-4^ | 0.71 |
| chr15 | 74426901 | 74427000 | ISLR2 | 5.33 x 10^-6^ | 0.71 |
| chr2 | 91635201 | 91635300 | NA | 1.02 x 10^-6^ | 0.71 |
| chr20 | 61637301 | 61637400 | BHLHE23 | 1.23 x 10^-5^ | 0.71 |
| chr5 | 72678101 | 72678200 | NA | 6.15 x 10^-4^ | 0.70 |
| chr10 | 28034001 | 28034100 | MKX | 4.88 x 10^-5^ | 0.70 |
| chr7 | 127672101 | 127672200 | LRRC4 | 1.75 x 10^-4^ | 0.70 |
| chr7 | 155167401 | 155167500 | NA | 8.21 x 10^-4^ | 0.70 |
| chr7 | 93519901 | 93520000 | TFPI2 | 1.64 x 10^-4^ | 0.70 |
| chr8 | 54789601 | 54789700 | RGS20 | 5.23 x 10^-4^ | 0.70 |
| chr18 | 74963301 | 74963400 | GALR1 | 2.11 x 10^-4^ | 0.70 |
| chr7 | 64974301 | 64974400 | NA | 4.25 x 10^-5^ | 0.70 |
| chr1 | 119527601 | 119527700 | TBX15 | 1.69 x 10^-4^ | 0.70 |
| chr13 | 28498401 | 28498500 | PDX1 | 6.11 x 10^-4^ | 0.70 |
| chr21 | 38069901 | 38070000 | SIM2 | 1.56 x 10^-4^ | 0.70 |
| chr21 | 38081001 | 38081100 | SIM2 | 2.52 x 10^-4^ | 0.69 |
| chr19 | 2252001 | 2252100 | AMH | 7.89 x 10^-5^ | 0.69 |
| chr9 | 122132101 | 122132200 | BRINP1 | 4.23 x 10^-5^ | 0.69 |
| chr1 | 146550701 | 146550800 | NA | 2.74 x 10^-4^ | 0.69 |
| chr13 | 113764501 | 113764600 | F7 | 7.37 x 10^-4^ | 0.69 |
| chr12 | 108238401 | 108238500 | NA | 3.13 x 10^-4^ | 0.69 |
| chr12 | 111472901 | 111473000 | CUX2 | 1.47 x 10^-7^ | 0.69 |
| chr5 | 72677801 | 72677900 | NA | 2.58 x 10^-5^ | 0.69 |
| chr20 | 61809301 | 61809400 | MIR124-3 | 8.09 x 10^-4^ | 0.68 |
| chr6 | 108486101 | 108486200 | NR2E1 | 1.91 x 10^-5^ | 0.68 |
| chr1 | 221050701 | 221050800 | HLX | 9.89 x 10^-6^ | 0.68 |
| chr20 | 26189601 | 26189700 | MIR663AHG | 1.19 x 10^-4^ | 0.68 |
| chr8 | 97506101 | 97506200 | SDC2 | 1.38 x 10^-4^ | 0.68 |
| chr9 | 19789201 | 19789300 | SLC24A2 | 8.43 x 10^-5^ | 0.68 |
| chr12 | 29937101 | 29937200 | TMTC1 | 2.05 x 10^-4^ | 0.68 |

**Table S1B. Top50 hypomethylated DMRs in CRC versus NAT comparison**

| **chr** | **start** | **stop** | **gene**  **symbol** | **p value** | **Δβ**  **(CRC-NAT)** |
| --- | --- | --- | --- | --- | --- |
| chr11 | 105642901 | 105643000 | GRIA4 | 2.52 x 10^-5^ | -0.74 |
| chr22 | 48604801 | 48604900 | NA | 4.97 x 10^-6^ | -0.74 |
| chr6 | 1426201 | 1426300 | NA | 2.72 x 10^-7^ | -0.73 |
| chr10 | 130396701 | 130396800 | NA | 2.52 x 10^-8^ | -0.73 |
| chrX | 141292401 | 141292500 | MAGEC2 | 1.11 x 10^-6^ | -0.72 |
| chr18 | 56652701 | 56652800 | ZNF532 | 5.92 x 10^-6^ | -0.71 |
| chr1 | 197128701 | 197128800 | ZBTB41 | 1.64 x 10^-7^ | -0.71 |
| chr3 | 117557501 | 117557600 | NA | 9.05 x 10^-5^ | -0.70 |
| chr7 | 18833801 | 18833900 | HDAC9 | 2.45 x 10^-4^ | -0.70 |
| chr5 | 120077301 | 120077400 | NA | 1.30 x 10^-4^ | -0.69 |
| chr4 | 43346201 | 43346300 | NA | 4.00 x 10^-7^ | -0.69 |
| chr11 | 17767901 | 17768000 | KCNC1 | 6.97 x 10^-6^ | -0.69 |
| chr3 | 116022801 | 116022900 | LSAMP | 7.96 x 10^-7^ | -0.69 |
| chr2 | 213272101 | 213272200 | ERBB4 | 4.62 x 10^-8^ | -0.69 |
| chr8 | 112078001 | 112078100 | NA | 4.29 x 10^-9^ | -0.68 |
| chr10 | 19907801 | 19907900 | MALRD1 | 3.12 x 10^-8^ | -0.68 |
| chr5 | 142727701 | 142727800 | NR3C1 | 6.28 x 10^-6^ | -0.68 |
| chr14 | 69754201 | 69754300 | GALNT16 | 7.09 x 10^-7^ | -0.68 |
| chr8 | 5094201 | 5094300 | NA | 2.48 x 10^-5^ | -0.68 |
| chr9 | 9395701 | 9395800 | PTPRD | 4.67 x 10^-7^ | -0.68 |
| chr4 | 22948401 | 22948500 | NA | 2.64 x 10^-7^ | -0.67 |
| chr1 | 108519601 | 108519700 | VAV3-AS1 | 1.35 x 10^-8^ | -0.67 |
| chr8 | 130125401 | 130125500 | NA | 9.51 x 10^-6^ | -0.67 |
| chr5 | 2129201 | 2129300 | NA | 9.71 x 10^-5^ | -0.67 |
| chr18 | 72595601 | 72595700 | ZNF407 | 2.13 x 10^-4^ | -0.67 |
| chr4 | 190607601 | 190607700 | NA | 5.14 x 10^-5^ | -0.67 |
| chr16 | 6250301 | 6250400 | RBFOX1 | 2.28 x 10^-5^ | -0.67 |
| chr21 | 29256801 | 29256900 | NA | 1.27 x 10^-6^ | -0.67 |
| chr1 | 106275201 | 106275300 | NA | 1.18 x 10^-7^ | -0.66 |
| chr17 | 45857801 | 45857900 | NA | 1.77 x 10^-4^ | -0.66 |
| chr21 | 44617901 | 44618000 | NA | 9.81 x 10^-6^ | -0.66 |
| chr6 | 138424501 | 138424600 | PERP | 3.60 x 10^-4^ | -0.66 |
| chr11 | 86726801 | 86726900 | NA | 3.33 x 10^-5^ | -0.66 |
| chr16 | 51033701 | 51033800 | NA | 6.73 x 10^-5^ | -0.66 |
| chr10 | 20707701 | 20707800 | NA | 7.67x 10^-10^ | -0.66 |
| chrX | 53004401 | 53004500 | FAM156A | 1.28 x 10^-6^ | -0.66 |
| chr7 | 28360501 | 28360600 | CREB5 | 7.82 x 10^-7^ | -0.66 |
| chr4 | 46319301 | 46319400 | GABRA2 | 9.46 x 10^-7^ | -0.65 |
| chr17 | 34339801 | 34339900 | NA | 1.94 x 10^-6^ | -0.65 |
| chr14 | 38576001 | 38576100 | NA | 3.39 x 10^-4^ | -0.65 |
| chr20 | 12186601 | 12186700 | NA | 9.50 x 10^-4^ | -0.65 |
| chr14 | 67853401 | 67853500 | NA | 2.66 x 10^-4^ | -0.65 |
| chr5 | 78077501 | 78077600 | ARSB | 7.57 x 10^-8^ | -0.65 |
| chr5 | 110494601 | 110494700 | NA | 2.68 x 10^-7^ | -0.65 |
| chr3 | 95433201 | 95433300 | NA | 1.27 x 10^-8^ | -0.65 |
| chr17 | 62045501 | 62045600 | SCN4A | 1.12 x 10^-4^ | -0.65 |
| chr5 | 159071601 | 159071700 | NA | 2.91 x 10^-6^ | -0.65 |
| chr18 | 76198901 | 76199000 | NA | 1.33 x 10^-4^ | -0.65 |
| chr8 | 64537701 | 64537800 | NA | 2.78 x 10^-8^ | -0.65 |
| chr12 | 41293401 | 41293500 | CNTN1 | 8.14 x 10^-5^ | -0.65 |

**Table S1C. Top50 hypermethylated DMRs in adenoma tissue compared to NAT samples**

| **chr** | **start** | **stop** | **gene symbol** | **p value** | **Δβ**  **(AD-NAT)** |
| --- | --- | --- | --- | --- | --- |
| chr10 | 106400501 | 106400600 | SORCS3 | 1.75 x 10^-12^ | 0.86 |
| chr10 | 8079301 | 8079400 | NA | 6.06 x 10^-10^ | 0.86 |
| chr2 | 131720701 | 131720800 | ARHGEF4 | 2.30 x 10^-12^ | 0.86 |
| chr16 | 86612401 | 86612500 | FOXL1 | 4.92 x 10^-12^ | 0.84 |
| chr6 | 84419201 | 84419300 | SNAP91 | 1.53 x 10^-8^ | 0.84 |
| chr14 | 63512501 | 63512600 | KCNH5 | 2.45 x 10^-6^ | 0.84 |
| chr19 | 58521201 | 58521300 | NA | 1.96 x 10^-11^ | 0.84 |
| chr22 | 19138401 | 19138500 | GSC2 | 1.92 x 10^-9^ | 0.83 |
| chr10 | 28034001 | 28034100 | MKX | 2.37 x 10^-9^ | 0.83 |
| chr11 | 134146101 | 134146200 | GLB1L3 | 2.45 x 10^-6^ | 0.83 |
| chr11 | 128562901 | 128563000 | FLI1 | 1.07 x 10^-9^ | 0.83 |
| chr3 | 58572601 | 58572700 | FAM107A | 2.84 x 10^-6^ | 0.83 |
| chr19 | 2252001 | 2252100 | AMH | 2.57 x 10^-7^ | 0.83 |
| chr13 | 28498401 | 28498500 | PDX1 | 1.51 x 10^-6^ | 0.83 |
| chr1 | 13839701 | 13839800 | LRRC38 | 3.53 x 10^-7^ | 0.83 |
| chr17 | 37381201 | 37381300 | STAC2 | 2.75 x 10^-7^ | 0.82 |
| chr1 | 63785501 | 63785600 | NA | 1.59 x 10^-6^ | 0.82 |
| chr15 | 89911201 | 89911300 | MIR9-3 | 3.23 x 10^-9^ | 0.82 |
| chr5 | 1877901 | 1878000 | IRX4 | 2.80 x 10^-10^ | 0.82 |
| chr1 | 3567901 | 3568000 | TP73 | 9.70 x 10^-11^ | 0.82 |
| chr4 | 1398301 | 1398400 | NKX1-1 | 1.11 x 10^-7^ | 0.81 |
| chr1 | 20879601 | 20879700 | FAM43B | 1.74 x 10^-6^ | 0.81 |
| chr8 | 11567101 | 11567200 | GATA4 | 2.79 x 10^-6^ | 0.81 |
| chr17 | 47574201 | 47574300 | NGFR | 1.74 x 10^-7^ | 0.81 |
| chr14 | 48143601 | 48143700 | MDGA2 | 2.11 x 10^-11^ | 0.81 |
| chr2 | 105459701 | 105459800 | LINC01158 | 4.11 x 10^-7^ | 0.81 |
| chr1 | 3568001 | 3568100 | TP73 | 4.48 x 10^-9^ | 0.81 |
| chr17 | 8907501 | 8907600 | NA | 5.30 x 10^-7^ | 0.80 |
| chr18 | 11752501 | 11752600 | GNAL | 7.63 x 10^-9^ | 0.80 |
| chr1 | 63785301 | 63785400 | NA | 2.56 x 10^-6^ | 0.80 |
| chr2 | 100937901 | 100938000 | LONRF2 | 2.28 x 10^-5^ | 0.80 |
| chr16 | 51184701 | 51184800 | SALL1 | 4.56 x 10^-8^ | 0.80 |
| chr10 | 23463201 | 23463300 | NA | 4.58 x 10^-7^ | 0.80 |
| chr14 | 51561201 | 51561300 | TRIM9 | 1.56 x 10^-7^ | 0.80 |
| chr10 | 94834701 | 94834800 | CYP26A1 | 1.51 x 10^-7^ | 0.80 |
| chr4 | 185942501 | 185942600 | NA | 3.98 x 10^-7^ | 0.80 |
| chr6 | 108486101 | 108486200 | NR2E1 | 5.96 x 10^-10^ | 0.80 |
| chr4 | 110223701 | 110223800 | COL25A1 | 1.84 x 10^-6^ | 0.80 |
| chr2 | 182548701 | 182548800 | NA | 3.59 x 10^-7^ | 0.79 |
| chr4 | 175750301 | 175750400 | GLRA3 | 1.43 x 10^-7^ | 0.79 |
| chr12 | 54441201 | 54441300 | HOXC4 | 1.13 x 10^-7^ | 0.79 |
| chr10 | 83634401 | 83634500 | NRG3 | 1.93 x 10^-5^ | 0.79 |
| chr16 | 86612501 | 86612600 | FOXL1 | 3.54 x 10^-9^ | 0.79 |
| chr17 | 8907301 | 8907400 | NA | 1.95 x 10^-7^ | 0.79 |
| chr20 | 43439501 | 43439600 | RIMS4 | 9.46 x 10^-8^ | 0.79 |
| chr12 | 117470801 | 117470900 | NA | 2.42 x 10^-6^ | 0.79 |
| chr17 | 27332501 | 27332600 | SEZ6 | 4.78 x 10^-7^ | 0.79 |
| chr1 | 17019401 | 17019500 | ESPNP | 3.05 x 10^-7^ | 0.79 |
| chr7 | 155167401 | 155167500 | NA | 5.65 x 10^-7^ | 0.79 |
| chr4 | 55098301 | 55098400 | PDGFRA | 3.01 x 10^-7^ | 0.79 |

**Table S1D. Top50 hypomethylated DMRs in adenoma tissue compared to NAT samples**

| **chr** | **start** | **stop** | **gene symbol** | **p value** | **Δβ**  **(AD-NAT)** |
| --- | --- | --- | --- | --- | --- |
| chrY | 6403901 | 6404000 | NA | 9.53 x 10^-14^ | -0.90 |
| chr16 | 60681101 | 60681200 | NA | 9.61 x 10^-10^ | -0.88 |
| chrY | 6404301 | 6404400 | NA | 7.37 x 10^-8^ | -0.88 |
| chr5 | 100645601 | 100645700 | NA | 5.38 x 10^-10^ | -0.85 |
| chrY | 6405301 | 6405400 | NA | 7.18 x 10^-11^ | -0.85 |
| chr5 | 117876101 | 117876200 | NA | 4.40 x 10^-7^ | -0.83 |
| chr5 | 135812801 | 135812900 | NA | 5.07 x 10^-8^ | -0.82 |
| chr4 | 108229701 | 108229800 | NA | 6.15 x 10^-11^ | -0.81 |
| chr12 | 60065801 | 60065900 | SLC16A7 | 4.00 x 10^-10^ | -0.81 |
| chrX | 80566101 | 80566200 | NA | 6.00 x 10^-9^ | -0.81 |
| chrX | 43658101 | 43658200 | MAOB | 1.72 x 10^-9^ | -0.81 |
| chr6 | 78312501 | 78312600 | NA | 9.23 x 10^-10^ | -0.80 |
| chr4 | 179184901 | 179185000 | NA | 3.05 x 10^-9^ | -0.80 |
| chr6 | 78312401 | 78312500 | NA | 5.12 x 10^-12^ | -0.80 |
| chr15 | 33069801 | 33069900 | FMN1 | 6.11 x 10^-11^ | -0.80 |
| chr2 | 185831901 | 185832000 | NA | 4.63 x 10^-10^ | -0.79 |
| chrX | 138585601 | 138585700 | NA | 8.76 x 10^-8^ | -0.79 |
| chrY | 6404901 | 6405000 | NA | 3.86 x 10^-5^ | -0.79 |
| chr8 | 5627601 | 5627700 | NA | 1.47 x 10^-11^ | -0.79 |
| chr2 | 142606901 | 142607000 | LRP1B | 5.61 x 10^-11^ | -0.79 |
| chr6 | 61970701 | 61970800 | NA | 1.74 x 10^-8^ | -0.79 |
| chr5 | 30353401 | 30353500 | NA | 1.80 x 10^-9^ | -0.78 |
| chr8 | 47900801 | 47900900 | NA | 3.95 x 10^-9^ | -0.78 |
| chrX | 113504601 | 113504700 | NA | 6.92 x 10^-7^ | -0.78 |
| chrY | 6404401 | 6404500 | NA | 7.22 x 10^-6^ | -0.78 |
| chrX | 80566001 | 80566100 | NA | 9.68 x 10^-8^ | -0.77 |
| chr13 | 57279601 | 57279700 | NA | 2.18 x 10^-6^ | -0.77 |
| chr4 | 71486301 | 71486400 | NA | 4.44 x 10^-10^ | -0.77 |
| chrX | 16395601 | 16395700 | NA | 1.38 x 10^-8^ | -0.77 |
| chrX | 16395901 | 16396000 | NA | 4.96 x 10^-7^ | -0.77 |
| chr7 | 49574401 | 49574500 | NA | 3.47 x 10^-6^ | -0.76 |
| chrY | 6405001 | 6405100 | NA | 4.39 x 10^-5^ | -0.76 |
| chrX | 105552601 | 105552700 | NA | 1.11 x 10^-8^ | -0.76 |
| chrX | 138585501 | 138585600 | NA | 1.21 x 10^-8^ | -0.76 |
| chrY | 24386701 | 24386800 | NA | 3.8 x 10^-9^ | -0.76 |
| chrY | 24386601 | 24386700 | NA | 1.91 x 10^-8^ | -0.76 |
| chr3 | 78451001 | 78451100 | NA | 9.91 x 10^-8^ | -0.76 |
| chr11 | 105642901 | 105643000 | GRIA4 | 1.08 x 10^-7^ | -0.76 |
| chr3 | 8741301 | 8741400 | NA | 2.74 x 10^-5^ | -0.75 |
| chr9 | 31621101 | 31621200 | NA | 4.52 x 10^-6^ | -0.75 |
| chr5 | 116877301 | 116877400 | LOC728342 | 3.89 x 10^-5^ | -0.75 |
| chr6 | 91674001 | 91674100 | NA | 3.52 x 10^-7^ | -0.75 |
| chrY | 6404701 | 6404800 | NA | 1.91 x 10^-5^ | -0.75 |
| chr1 | 63009301 | 63009400 | DOCK7 | 2.10 x 10^-7^ | -0.75 |
| chr12 | 40107301 | 40107400 | C12orf40 | 5.95 x 10^-6^ | -0.75 |
| chr10 | 33794301 | 33794400 | NA | 7.11 x 10^-9^ | -0.75 |
| chrX | 16395701 | 16395800 | NA | 5.94 x 10^-7^ | -0.74 |
| chr3 | 95502901 | 95503000 | NA | 1.07 x 10^-5^ | -0.74 |
| chr6 | 102912001 | 102912100 | NA | 3.32 x 10^-5^ | -0.74 |
| chrX | 88463401 | 88463500 | NA | 1.39 x 10^-7^ | -0.74 |
