# Supplementary material for: Gene promoter and exon DNA methylation changes in colon cancer development – mRNA expression and tumor mutation alterations
Source: BMC Cancer. 2018 Jun 27;18:695. doi: 10.1186/s12885-018-4609-x (PMC6020382; doi:10.1186/s12885-018-4609-x)

**Figure S1. Box plots of significant DMRs in *TP53* pathway gene promoters between CRC and NAT samples**

Box plots represent the DNA methylation levels (β-values) of significant differentially methylated regions (DMRs) in *TP53* pathway gene promoters. Individual DNA methylation level values are shown by red dots, and the median and standard deviation of the β-values are also demonstrated**.** The names of the DMRs indicate the official gene symbol_number of the chromosome_start position of the DMR. NAT = normal adjacent tissue**;** CRC = colorectal cancer; ATR = ATR serine/threonine kinase; CASP8 = caspase 8; CCNE1 = cyclin E1; CDKN1A = cyclin dependent kinase inhibitor 1A; CDKN2A = cyclin dependent kinase inhibitor 2A; CYCS = cytochrome c, somatic; EI24 = EI24 autophagy associated transmembrane protein; FAS = Fas cell surface death receptor; GTSE1 = G2 and S-phase expressed 1; IGF1 = insulin like growth factor 1; IGFBP3 = insulin-like growth factor binding protein 3; PTEN = phosphatase and tensin homolog; RFWD2 = ring finger and WD repeat domain 2; RRM2 = ribonucleotide reductase regulatory subunit M2; SESN2 = sestrin 2; SESN3 = sestrin 3; THBS1 = thrombospondin 1; TP73 = tumor protein p73

**Figure S2. DNA methylation pattern of top50 hypermethylated DMRs on an independent set of samples**

BeadChip450K array CpG sites located on the top50 significantly hypermethylated DMRs in our methyl capture sequencing study were evaluated in CRC vs. NAT and AD vs. NAT comparisons. **A.** GSE48684: CRC vs. NAT; **B.** GSE48684: AD vs. NAT; **C.** The Cancer Genome Atlas: CRC vs. NAT. On heatmaps, samples are represented in columns, CpG sites with Illumina Beadchip450K cg IDs are shown in rows. Hypermethylation is marked with red, while hypomethylated CpG sites are green. CRC = colorectal cancer, Ad = adenoma, NAT = normal adjacent tissue

**
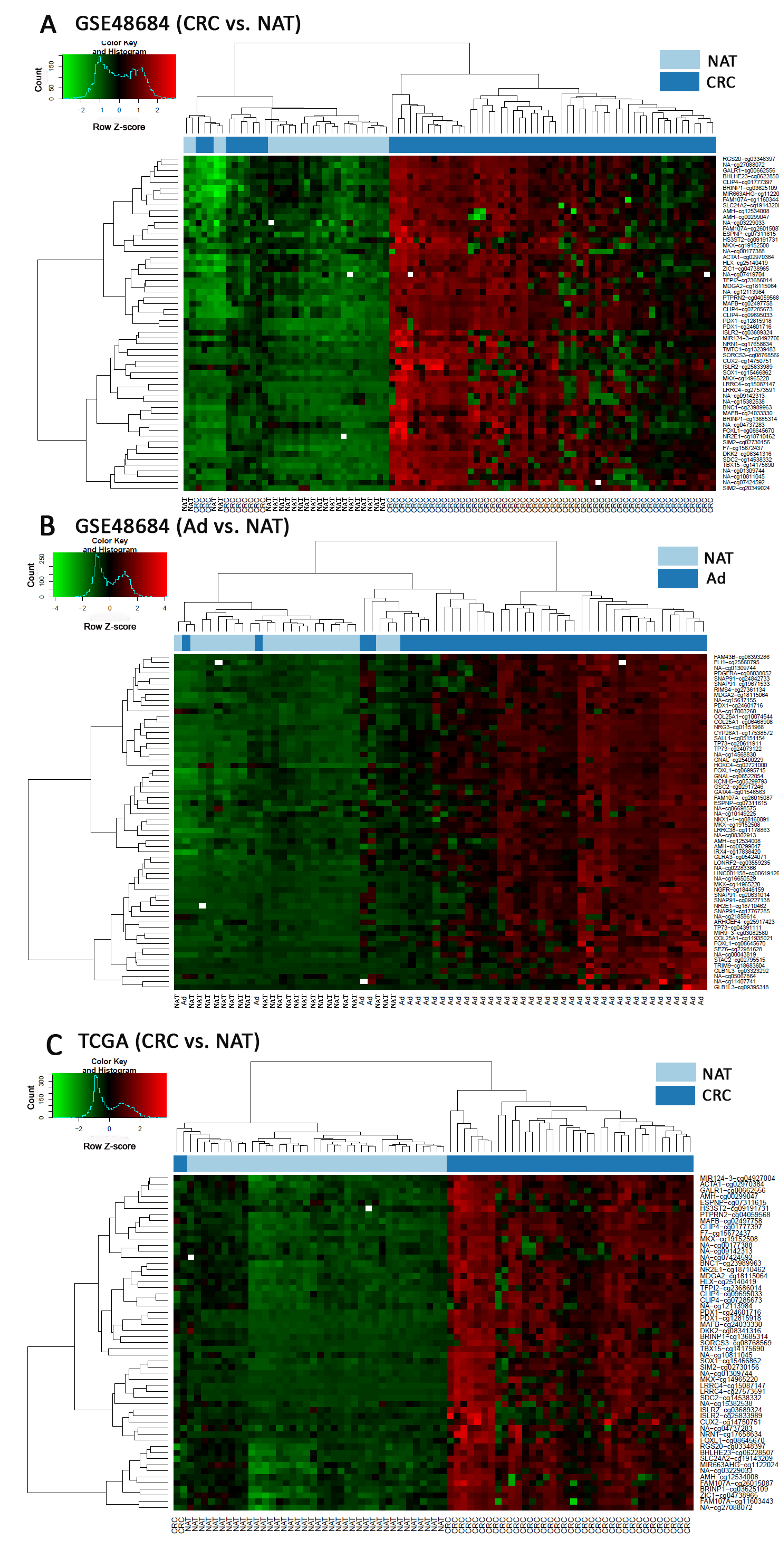
**

**Figure S3. DNA methylation profiles of the overlapping regions on methyl capture sequencing and EpiTect array results**

**A.** CRC vs. NAT MethylCap-Seq**; B.** CRC vs. NAT EpiTect Methyl array **C.** AD vs. NAT MethylCap-Seq **D.** AD vs. NAT Epitect Methyl array. On heatmaps, samples are represented in columns, significantly differentially methylated regions (DMRs) are shown in rows (p<0.05). Hypermethylation is marked with red, while hypomethylated DMRs are green. The overlapping samples are numbered on heatmaps. CRC = colorectal cancer, AD = adenoma, NAT = normal adjacent tissue


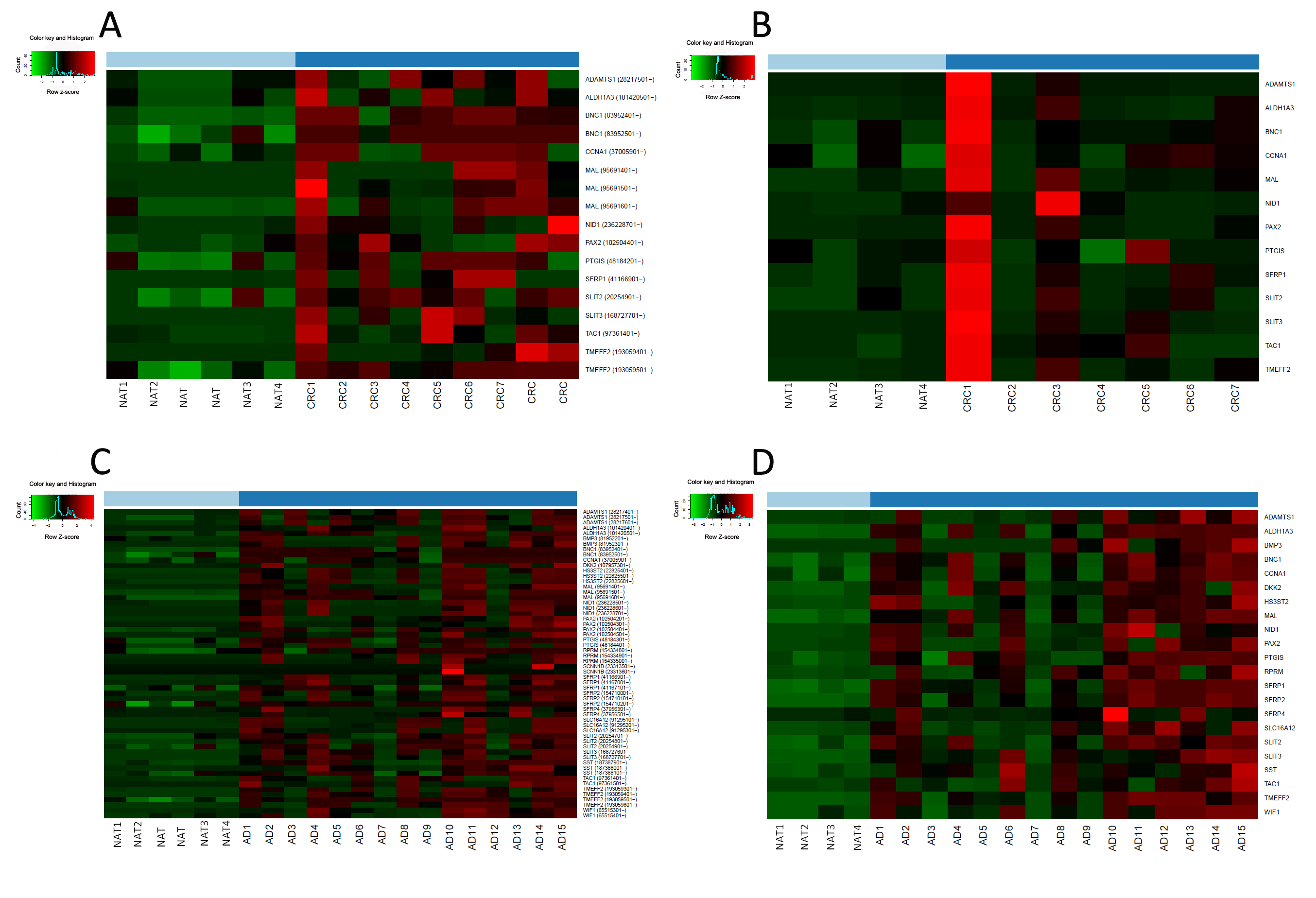

Supplement: Supplementary file 2 — Figure S1. Box plots of significant DMRs in TP53 pathway gene promoters between CRC and NAT samples. Figure S2. DNA methylation pattern of top50 hypermethylated DMRs on an independent set of samples. Figure S3. DNA methylation profiles of the overlapping regions on methyl capture sequencing and EpiTect array results. (DOC 902 kb) [file 12885_2018_4609_MOESM2_ESM.doc]
